# Inequities in accessibility to and utilisation of maternal health services in Ghana after user-fee exemption: a descriptive study

**DOI:** 10.1186/s12939-014-0089-z

**Published:** 2014-11-01

**Authors:** John K Ganle, Michael Parker, Raymond Fitzpatrick, Easmon Otupiri

**Affiliations:** The Ethox Centre, Nuffield Department of Population Health, University of Oxford, Rosemary Rue Building, Old Road Campus, Headington, Oxford OX3 7LF UK; Nuffield Department of Population Health, University of Oxford, Rosemary Rue Building, Old Road Campus, Headington, Oxford OX3 7LF UK; Department of Community Health, School of Medical Sciences, Kwame Nkrumah University of Science and Technology, Kumasi, Ghana

**Keywords:** User-fee exemption, Maternal health, Access, Inequity, Ghana

## Abstract

**Introduction:**

Inequities in accessibility to, and utilisation of maternal healthcare services impede progress towards attainment of the maternal health-related Millennium Development Goals. The objective of this study is to examine the extent to which maternal health services are utilised in Ghana, and whether inequities in accessibility to and utilization of services have been eliminated following the implementation of a user-fee exemption policy, that aims to reduce financial barriers to access, reduce inequities in access, and improve access to and use of birthing services.

**Methods:**

We analyzed data from the 2007 Ghana Maternal Health Survey for inequities in access to and utilization of maternal health services. In measuring the inequities, frequency tables and cross-tabulations were used to compare rates of service utilization by region, residence and selected socio-demographic variables.

**Results:**

Findings show marginal increases in accessibility to and utilisation of skilled antenatal, delivery and postnatal care services following the policy implementation (2003–2007). However, large gradients of inequities exist between geographic regions, urban and rural areas, and different socio-demographic, religious and ethnic groupings. More urban women (40%) than rural, 53% more women in the highest wealth quintile than women in the lowest, 38% more women in the best performing region (Central Region) than the worst (Upper East Region), and 48% more women with at least secondary education than those with no formal education, accessed and used all components of skilled maternal health services in the five years preceding the survey. Our findings raise questions about the potential equity and distributional benefits of Ghana’s user-fee exemption policy, and the role of non-financial barriers or considerations.

**Conclusion:**

Exempting user-fees for maternal health services is a promising policy option for improving access to maternal health care, but might be insufficient on its own to secure equitable access to maternal health services in Ghana. Ensuring equity in access will require moving beyond user-fee exemption to addressing wider issues of supply and demand factors and the social determinants of health, including redistributing healthcare resources and services, and redressing the positional vulnerability of women in their communities.

## Introduction

Lack of access and unequal access to essential maternal healthcare services have been identified as the main underlying causes of maternal deaths across the world, but specifically in Sub-Saharan Africa [1-5]. There is evidence to suggest that access to appropriate healthcare, especially skilled attendance at birth and timely referrals to emergency obstetric care, is strongly associated with substantial reductions in mortality and morbidity for both mother and newborn [6-9]. However, in many countries of sub-Saharan Africa including Ghana, few women use health facilities for birth [9]. While in high-income countries coverage of skilled birthing services is almost universal, in Africa only 47% of women give birth with a skilled care provider [7].

In Ghana, maternal mortality is the second largest cause of female deaths, and accounts for 14% of all female deaths [9]. In 2012, the World Health Organization (WHO) estimated that Ghana’s maternal mortality ratio (MMR) was 350 maternal deaths per 100, 000 live births [10]. In addition, large and growing gradients of inequities in service accessibility and utilization have also been observed [11].

In an effort to achieve the Millennium Development Goal (MDG) 5 the government of Ghana introduced and is currently implementing a policy that provides free maternal health services to all women in all government, mission, and selected private health facilities [11,12]. The policy was first introduced in the 4 most deprived regions of the country (Northern, Upper East, Upper West, and Central) in 2003, and later extended to Ghana’s remaining 6 regions in 2005 [13]. Under the policy, all women are entitled to a ‘Maternal Benefit Package’, that includes 6 free antenatal visits; additional medically necessary visits captured as out patient department visits; free delivery at a health facility, including all delivery-related complications; 2 postnatal visits within 6 weeks; and care for the newborn up to three months.

The main argument in support of Ghana’s user-fee exemption policy is that financial costs are a major barrier to skilled care and that the poor would not be able to afford to pay for the use of necessary services. The policy is therefore expected to reduce both the financial barriers to access and inequities in access, particularly access to supervised delivery services [14]. Indeed, within Sub-Saharan Africa, more than 11 other countries including Senegal, Burkina Faso, Mali, Kenya, Niger, and Tanzania have implemented similar policies [15-22]. Outside Africa, Nepal, Cambodia, China, Bangladesh, India, Pakistan and Bolivia are also implementing various cash transfer and user-fee exemption programmes for skilled maternal health services [15,23-28].

Despite the popularity of this new policy intervention, it is not clear to what extent skilled maternal healthcare services have become widely accessible and used in Ghana. It is also not clear whether variations in accessibility to and utilization of skilled care have been eliminated following the implementation of the policy in Ghana. To the authors’ knowledge, evaluative studies of Ghana’s user-fee exemption policy [11,12] have not examined the equity dimension of access. One recent study observed that rigorous evaluations of whether the policy ensures universal access by eliminating inequities in access and service utilization are lacking [29]. The objective of this paper is to assess Ghana’s user-fee exemption policy from an equity perspective, describing and exploring the extent to which it eliminates inequities in access to and use of maternal health services.

## Materials and methods

### Study design

The study reported in this paper forms part of a larger, original study that the authors conducted to examine the effects of Ghana’s user-fee exemption policy on women’s maternity care seeking experience, equity of access, and barriers to accessibility and utilization of maternal and newborn healthcare services. The design of this larger study followed a mixed methods approach; involving analysis of a nationally representative retrospective household survey data in combination with qualitative exploration using data generated from anthropological research techniques of focus group discussions, in-depth interviews and structured field observations. In this paper, we focus on and report findings from the quantitative component of the study, which assessed inequities in accessibility to, and utilisation of maternal health services in Ghana.

### Study context

Ghana is a lower middle-income West African country, with an estimated total population of 24,658, 823 [30]. Average life expectancy at birth is 60 (59 for male and 60.7 for females). Adult literacy - defined as the proportion of population aged 15 years or above who can read and write in English and a Ghanaian language - is 57.9%. Ghana has a human development index (HDI) of 0.526 and a multi-dimensional poverty index of 0.14. In 2005, about 30% of Ghana’s population was estimated to live on less than US$1 per day.

Like many lower-income countries, communicable diseases account for about two-thirds of out patient department visits in Ghana, with malaria being the main cause of outpatient morbidity [31]. In addition to the fact that maternal health outcomes continue to be poor in Ghana, we chose Ghana for this research because it is one of only a handful of countries in Africa to have actively started implementing both universal maternity care and health insurance policies at the national level. Because of this, Ghana is often seen as ‘an example of global good practice’ [32]. Despite this, maternal, neonatal and infant mortality ratios have remained persistently high in Ghana.

### Data sources

The data for this study were extracted from the Ghana Maternal Health Survey 2007. The GMHS is the first nationally representative, high-quality population-based survey to collect information specifically on maternal health services accessibility and utilization since the implementation of the fee-free maternal health policy. The survey is a retrospective five-year (2003–2007) nationally representative survey of 10,858 households and 10,370 individual women aged 15–49 years. The survey was carried out to collect data to assess the level of maternal mortality in Ghana; identify specific causes of maternal and non-maternal deaths; and measure indicators of access to and utilization of maternal health services in Ghana.

The survey was conducted in two phases. In phase I, a short nationally representative household survey questionnaire was administered to 240,000 households from 1,600 clusters or primary sampling units within the 10 administrative regions of Ghana. The 1,600 clusters were selected from a pre-existing list created for Ghana’s 2000 Population and Housing Census. Out of the 240,000 households sampled in phase I, 226,209 households completed the questionnaire, with a 94.3% response rate. The purpose of the Phase I survey was to identify deaths to women aged 12–49 years in the 5 years preceding the survey. In Phase II, a verbal autopsy survey was conducted with households that reported one or more deaths of women aged 12–49 years. Apart from the verbal autopsy survey, Phase II also involved interviews with individual women aged 15–49 years from a total of 11,579 randomly selected households (independent of the households identified in Phase I as having experienced a female death). Of the 11,579 households, 10,994 were occupied at the time of the survey. However, 10,858 households were successfully interviewed, giving a response rate of 99%. From the 10,858 interviewed households, a total of 10,627 women were identified as eligible for individual interview (i.e. women aged 15–49 years). Interviews were however completed for 10,370 women - 98% response rate - using a questionnaire for individual women. The purpose of this Phase II survey was to collect information on key demographic and maternal and neonatal health indicators such as access and use of antenatal and emergency obstetric care in the event of a birth, abortion, or miscarriage. For the purposes of this paper, we used data from the interviews with individual women (i.e. data relating to access to and use of antenatal, maternity, and emergency obstetric care) generated in the second part of Phase II of the survey with the 10,370 individual women. Our analysis involved a total of 5,077 births – 4996 live births and 81 stillbirths – that were recorded in the five years preceding the survey.

### Measuring inequities

According to the International Society for Equity in Health, equity is the absence of potentially remediable, systematic differences in access and use of one or more aspects of maternal health services across socially, economically, demographically, or geographically defined population groups or subgroups [33]. This definition is useful for the discussion in this paper because it suggests that non-medical features of individuals or groups (such as their geographic location or ability to pay) should not determine their access to skilled maternity care services. It also implies a situation in which individuals or groups face equal or equivalent access and costs of utilization for equal or equivalent need [34].

In attempting to assess inequities in accessibility to, and utilisation of maternal health services, we used a three-step process outlined by Zere and colleagues [3,5,31]. These steps are: (i) identification of the care intervention whose distribution is to be measured; (ii) classification/grouping of the population into different strata by a selected equity stratifier; and (iii) measuring the degree of inequality.

#### The interventions

The first step in assessing inequities involved definition of the interventions whose distributions are to be measured. These interventions included antenatal check-ups, tetanus toxoid immunization, delivery at a health facility, skilled attendance at birth, and caesarean sections (CS) during delivery, and postnatal check-ups. Access and equity of access to antenatal care was assessed by the timing, number of visits and type of care provider, and measured by the percentage of women from different socio-demographic backgrounds receiving these types of services. We assessed inequities in protection against tetanus by comparing the percentage of women from different socio-demographic backgrounds receiving the WHO recommended doses of at least two tetanus toxoid injection during their last live or still birth in the five years preceding the survey. Inequities in access to, and use of delivery care was measured by skilled attendant at delivery (i.e. percentage of births delivered by skilled providers including doctor, nurse, midwife, auxiliary midwife and community health officer), delivery in a health facility (i.e. percentage of births delivered in a public or private sector health facilities), delivery at public facility (i.e. percentage of births delivered in public sector health facilities), and home delivery (i.e. percentage of births delivered at home). We assessed inequities in access to and use of CS by measuring the percentage of live births in the five years preceding the survey delivered by CS according to our variable stratifiers. Within the literature, there is debate about the acceptable level at which a given population should be receiving CS [35-38]. Recently however, it has been argued that the proportion of deliveries by CS in a geographical area is a measure of access to, and use of, obstetric emergency care for averting maternal and newborn mortality, and that a population-based rate of 5–15% is considered as the acceptable level of CS to ensure the best outcomes for mothers and newborns [3]. Finally, we assessed inequities in postnatal care access by comparing whether a woman sought care after delivery and from whom across our variable stratifiers.

#### The variables

In the second step, we classified women by variable stratifiers against which accessibility to and utilisation of antenatal, delivery and postnatal care services was then assessed. These variables were mother’s education, mother’s age at birth, birth order, place of residence (urban/rural), geographical region of residence, wealth quintiles, religion and ethnicity. The survey data we used do not contain data on household income or consumption income. Therefore wealth index is used as a proxy. This wealth index is constructed from household ownership of assets and consumer goods (radio, television, telephone and refrigerator), dwelling characteristics, type of drinking water source, toilet facilities, electricity, wall and floor materials of house, cooking fuel, and means of transport. Each asset was assigned a weight (factor score) generated using the methods of principal component analysis [5], and the resulting asset scores standardized in relation to a normal distribution with a mean of zero and standard deviation of one (see [31]. From here, each household was given a score for each asset and these asset scores were then summed up for each household. Finally, individual women were ranked according to the total score of the household they came from; the sample was then divided into quintiles from lowest (one) to highest (five). Following this, a single asset index was developed for the whole sample, with no separate indices prepared for different regional or urban and rural populations.

We acknowledge that gauging the wealth status of households based on assets may be flawed because ownership of consumer goods is partly a function of taste and choice, and may therefore be independent of wealth [39]. Research has however shown that household assets often approximate the long-run economic status of households [40].

#### Analytical method

In the third and final step, we assessed access patterns, and equity in utilisation of the interventions we defined in the first step by analysing and comparing accessibility and utilization rates across the variable stratifiers using descriptive statistical tools. Within the healthcare literature, there is still considerable debate regarding the development of appropriate methods for assessing inequities in health and differentials in access among social groups [41]. However, Gulliford’s recent work summarizes the different debates to suggest three main approaches, namely those depending on simple comparison of rates of access for different groups; those depending on the use of regression methods; and those that rely on the development of Gini-like coefficients [41]. Given that our study is mainly descriptive, we chose the first approach. Rates of access were compared for different population groups using both absolute measures (the difference in rates between the selected group and the reference group), and relative measures (the ratio of rates between selected and reference groups). We analysed all the data using the IBM SPSS Statistics data analysis software package (version 20), and MS Excel.

## Results

### Descriptive statistics

**Figure 1:**
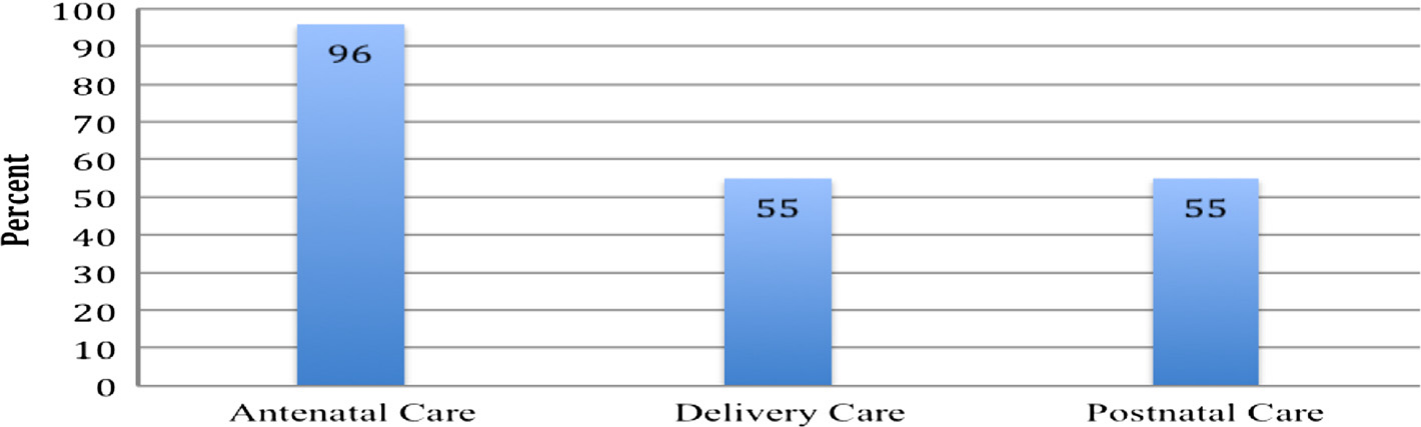
**Percentage distribution of coverage of ANC, DC and PNC by skilled provider for the most recent live or stillbirth in the 5 years preceding the survey.**

Figure 1 depicts the national coverage of access to, and use of antenatal care (ANC), delivery care (DC), and postnatal care (PNC) services in Ghana by skilled providers in percentage terms as at 2007 according to the GMHS.

At the national level, 96% of pregnant women in the five years preceding the survey (2003–2007) received at least one ANC from a skilled provider. In comparison with the baseline figure of 92% reported in Ghana’s 2003 Demographic and Health Survey, the number of births who received skilled ANC during the first five years (i.e. 2003–2007) of implementing the user-fee exemption policy increased by an average of 4%. The 96% recorded for skilled ANC however dramatically decreased to 55% each for skilled assistance during delivery and postnatal care following delivery. Compared with the baseline data in Ghana’s 2003 Demographic and Health Survey again, skilled attendance at delivery went up from 47% in 2003 to 55% in 2007. This represents a percentage change of 8%. Similar incremental changes are observed for tetanus toxoid immunisation during pregnancy, delivery in a health facility, CS, and postnatal check-up. For example, the percentage of pregnant women who received at least two dosages of tetanus toxoid protection increased from 50% in 2003 to 62% in 2007, while delivery in a health facility rose by 10% (i.e. from 46% in 2003 to 54% in 2007).

**Figure 2:**
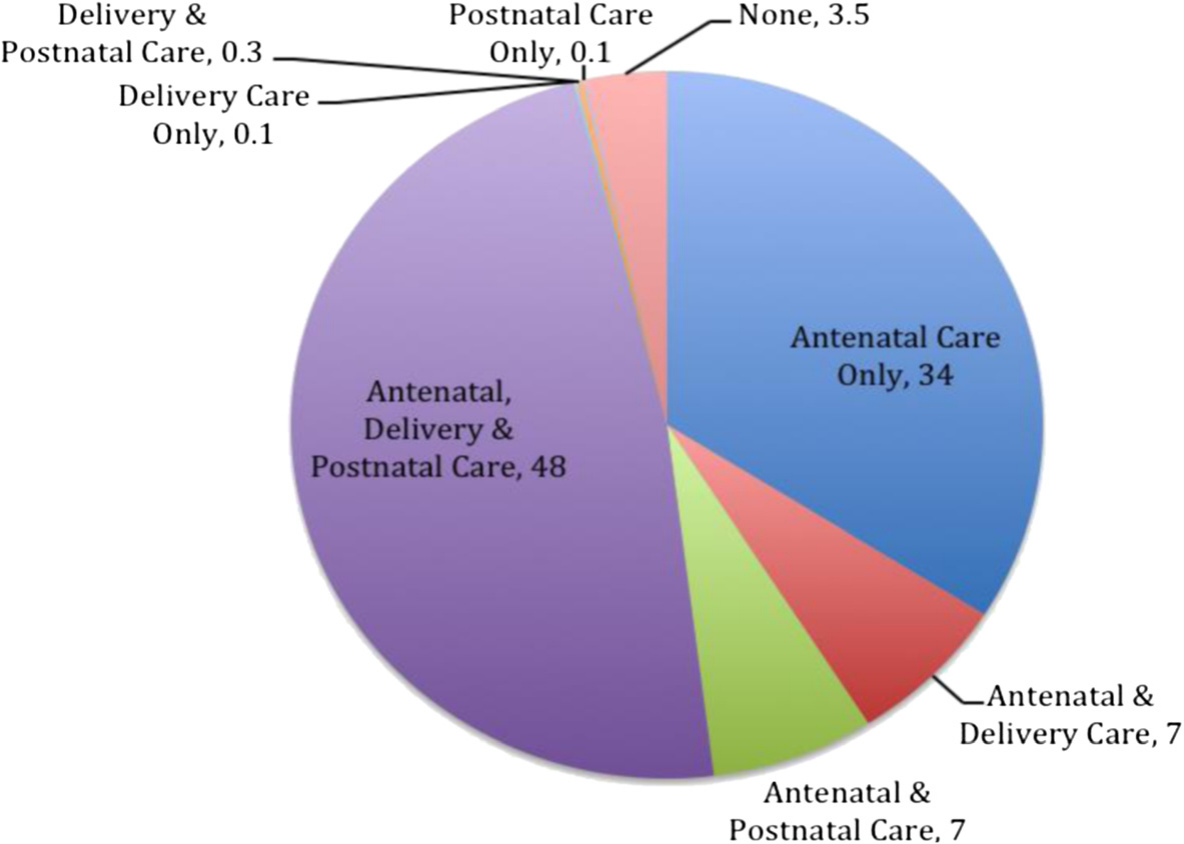
**Percentage distribution of completeness of access to and use of skilled maternity care services in Ghana.**

In terms of the distribution of access to and use of all components of maternal health services in the five years preceding the survey at the national level, only one-in-two women (48%) accessed and used all three maternity care components, i.e. ANC, DC and PNC (Figure 2). About 4% of women did not receive a single component of maternity care at all, while 34% of women received ANC only. Similarly, 7% of the women received both ANC and DC or ANC and PNC only.

**Figure 3:**
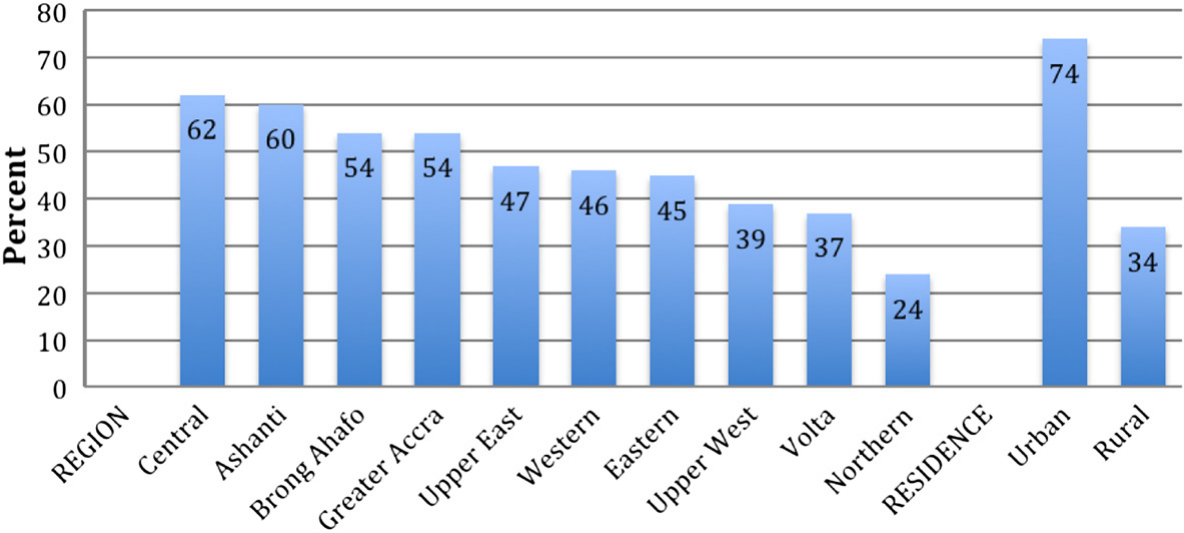
**Per cent distribution of most recent live or stillbirth in the five years preceding the survey for which skilled ANC, DC, and PNC were received by region and residence.**

**Figure 4:**
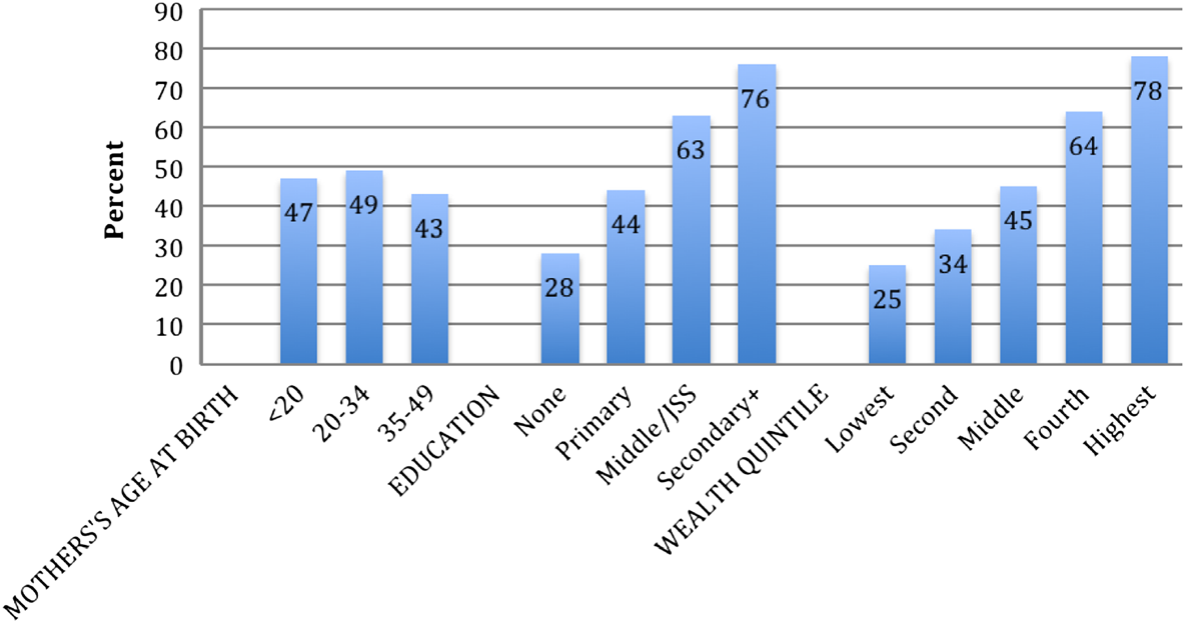
**Per cent distribution of most recent live birth or still birth in the 5 years preceding the survey for which ANC, DC & PNC were received by mother’s age at birth, level of education, and wealth.**

Although the above statistics are useful in giving a broad understanding of the levels of skilled maternity care coverage at the national level, what is not easily discernable through these national level statistics is that large gradients of inequities in complete coverage of maternity care service accessibility and utilization exist for the different categories of respondents (Figures 3 and 4). For example, less educated women, rural women and women in poorer households were less likely to receive complete maternity care than urban, more educated and wealthier women. Complete coverage of maternity care services also declines with birth order from 58% for first births to 34% among births of six or higher, and is highest among mothers aged 20–34 years. In comparative regional terms, complete coverage of maternity care ranged from a high of 62% in the Central region to a low of 24% in the Northern region. In other words, in the five years preceding the study, whereas 62% of all births in the Central region received all the three main maternity care components, only 24% of births in the Northern region did. But the inequities in completeness of maternity care access between geographic regions, urban and rural areas, and different socio-demographic groupings appear to be reproduced by inequities in the individual maternity care interventions. For this reason, the next sections focus on examining the nature of these access and utilisation inequities separately for ANC, tetanus immunization, DC, delivery at a health facility, skilled attendance at delivery, caesarean sections (CS), and PNC.

### Inequities in access and use of ANC services

Tables 1 and 2 show the distribution of ANC service accessibility and utilization by women who either had a live or stillbirth in the five years preceding the survey according to various background characteristics. Generally, inequities in levels of antenatal care access among subgroups of women in Ghana appear to be smaller, at least in the five years preceding the survey. Yet, as Table 1 shows, the percentage difference between antenatal care from a skilled provider (doctor) for the best performing region (Greater Accra, 46%) and the worst (Upper East, 2%) is 44%. ANC access was also more common among mothers who have had a live birth (96%) than among mothers who have had a stillbirth (88%), and is highest among births to mothers aged 20 years or below and among first order births (Table 1).

**Table 1:** **Per cent distribution of women who had a live or stillbirth in the five years preceding the survey by whether mother received ANC from a skilled provider**

**Background characteristic**	**Received any ANC**	**Doctor**	**Nurse/midwife/auxiliary midwife**	**Trained traditional birth attendant**	**Untrained traditional birth attendant**	**Other**	**No one**	**Missing**	**Total**	**Percent receiving ANC from a skilled provider**	**Number of women**
**Birth outcome**											
Live birth	96.7	18.5	77.8	0.2	0.1	0.1	3.2	0.1	100	96.3	4,847
Stillbirth	87.9	23.1	64.7	0	0	0	10.2	2	100	87.9	81
**Age of mother at birth**											
<20	97	14.7	82.1	0.1	0	0	3	0	100	96.8	534
20–34	96.8	19.2	77.2	0.2	0.1	0.1	3	0.1	100	96.4	3,391
35–49	95.2	18.1	76.6	0.3	0.1	0	4.4	0.4	100	95	1,003
**Birth order**											
1	98.6	20.7	77.7	0.1	0	0.2	1.2	0.2	100	98.4	1,051
2–3	97.2	20.5	76.4	0.2	0	0	2.7	0.1	100	96.9	1,769
4–5	96.9	18.3	77.8	0.2	0.4	0.2	2.9	0.2	100	96.1	1,161
6+	92.4	12.7	79.4	0.2	0.1	0	7.3	0.2	100	92.1	948
**Residence**											
Rural	95.4	12.1	82.8	0.2	0.1	0	4.6	0	100	94.9	3,245
Urban	98.7	30.8	67.6	0.1	0.1	0	0.9	0.4	100	98.4	1,683
**Education**											
None	93.5	11.1	82.1	0.2	0.1	0.1	6.3	0.1	100	93.2	1,678
Primary	96.7	18.2	77.9	0.2	0.4	0.1	2.8	0.4	100	96.1	1,102
Middle/JSS	98.6	21.3	76.9	0.3	0	0	1.4	0	100	98.2	1,797
Secondary+	99.5	40.8	58.6	0	0	0.3	0.5	0	100	99.5	350
**Region**											
Ashanti	97.9	27.6	69.8	0	0.1	0	1.9	0.2	100	97.5	922
Brong Ahafo	98	11.4	86.3	0	0.3	0	2	0	100	97.7	564
Central	97.8	13.2	84.4	0.2	0	0.1	2.2	0	100	97.7	479
Eastern	97.2	16.9	79.4	0.7	0.2	0	2.7	0.1	100	96.2	567
Greater Accra	96.4	45.7	50.1	0.2	0.4	0	3.2	0.4	100	95.8	470
Northern	91.7	7.6	84	0.1	0	0	8.2	0.2	100	91.6	699
Volta	96.4	10.7	85.7	0	0	0	3.5	0.1	100	96.4	451
Upper East	98.7	2.4	95.9	0.5	0	0	1.3	0	100	98.3	225
Upper West	94.3	3.4	90.9	0	0	0	5.7	0	100	94.3	152
Western	97	27.2	69.2	0.7	0	0	2.5	0.5	100	96.3	400
**Wealth quintile**											
Lowest	93.1	7.9	84.8	0.2	0.3	0.1	6.9	0	100	92.7	1,074
Second	94.9	9.4	85	0.3	0.1	0.1	5	0.1	100	94.4	1,061
Middle	98.2	16.2	81.7	0.3	0	0.2	1.8	0	100	97.9	975
Fourth	98.1	25.5	72.2	0.2	0	0	1.5	0.3	100	97.7	983
Highest	99	38.2	60.6	0	0.2	0.1	0.6	0.4	100	98.7	835
**Total**	**96.5**	**18.5**	**77.6**	**0.2**	**0.1**	**0.1**	**3.3**	**0.2**	**100**	**96.1**	**4,928**

**Table 2:** **Per cent distribution of number of ANC visits and median number of visits for the most recent live birth or stillbirth in the five years preceding the survey**

**Background characteristic**	**0**	**1**	**2–3**	**4+**	**Don’t know/missing**	**Total**	**Median number of visits**	**Number of births**
**Birth outcome**								
Live birth	3.2	3.4	16	77	0.5	100	5.9	4,847
Stillbirth	10.2	9.7	13.8	63.3	3.1	100	5.6	81
**Age of mother at birth**								
<20	3	3.9	20.9	71.7	0.5	100	5.2	534
20–34	3	3	14.9	78.5	0.5	100	6.1	3,391
35–49	4.4	4.8	16.6	73.3	0.8	100	5.7	1,003
**Birth order**								
1	1.2	2.9	14	81.5	0.4	100	6.2	1,051
2–3	2.7	2.9	14.2	79.6	0.5	100	6.1	1,769
4–5	2.9	3.4	16.2	76.8	0.6	100	6 5	1,161
6+	7.3	5.1	20.8	66	0.8	100	2	948
**Residence**								
Rural	4.6	4.5	20.3	70.3	0.4	100	5.3	3
Urban	0.9	1.5	7.5	89.1	1	100	7.3	1
**Education**								
None	6.3	5.2	19.2	68.6	0.8	100	5.3	1,678
Primary	2.8	4.4	21.1	70.5	1.1	100	5.4	1,102
Middle/JSS	1.4	1.7	11.8	85	0.1	100	6.5	1,797
Secondary+	0.5	1	5.2	92.9	0.4	100	7.9	350
**Region**								
Ashanti	1.9	3.1	11.5	83.2	0.3	100	6.6	922
Brong Ahafo	2	4.3	15.8	77.7	0.1	100	5.9	564
Central	2.2	3.1	10.1	84.3	0.2	100	6.2	479
Eastern	2.7	4.3	20.1	72.8	0.1	100	5.6	567
Greater Accra	3.2	1.9	12.2	80.9	1.7	100	7.5	470
Northern	8.2	4.7	14.8	71.6	0.8	100	5.5	699
Volta	3.5	5.9	29.9	60.6	0.1	100	4.8	451
Upper East	1.3	0	10.6	87.6	0.6	100	5.8	225
Upper West	5.7	2.1	20	68.9	3.5	100	5.9	152
Western	2.5	1.5	19.3	76.1	0.5	100	5.5	400
**Wealth quintile**								
Lowest	6.9	7.2	23.6	61.9	0.5	100	4.9	1,074
Second	5	4.3	20.8	69.4	0.5	100	5.2	1,061
Middle	1.8	2.6	18.7	76.7	0.2	100	5.5	975
Fourth	1.5	1.4	9.9	86.5	0.5	100	6.7	983
Highest	0.6	0.9	3.7	93.7	1.1	100	8.3	835
**Total**	**3.3**	**5.3**	**15.9**	**76.7**	**0.6**	**100**	**5.9**	**4,928**

Less surprisingly, the frequency of ANC visits is higher in urban than rural areas, with 89% of urban women seeking care at least 4 times, compared with 70% of rural women (Table 2). Differences by region ranged from a low median of 3.4 months in the Upper West to a high of 4.3 months in the Northern region for seeking initial antenatal care. Antenatal care access is also higher among women with secondary or higher level of education (7.9) than women with no education (5.3), and among those in the highest wealth quintile (8.3) than those in the lowest quintile (4.9).

### Inequities in access to tetanus toxoid immunization

Tables 3 and 4 show that nearly 62% of the women who took part in the survey received at least two doses of tetanus toxoid during pregnancy for their most recent birth. Similarly about four-in-five women (79%) were protected against tetanus for their last birth. There are however important differences in access and utilisation levels between different regions, wealth groups, and ethnic and religious groups (Table 3 and 4).

**Table 3:** **Tetanus toxoid immunization during pregnancy for the last birth in the five years preceding the survey**

**Background characteristic**	**Percent receiving two or more injections during last pregnancy**	**Percent whose last birth was protected against neonatal tetanus**	**Number of mothers**
**Birth outcome**			
Live birth	61.9	79.6	4,847
Stillbirth	47.3	69.4	81
**Mother’s age at birth**			
<20	59.5	72.4	534
20–30	62.2	80.4	3,391
35–49	61.1	79.9	1,003
**Birth order**			
0–1	66.2	73.8	1,051
2–3	60.4	81.4	1,769
4–5	60.3	81.8	1,161
6+	60.8	79.1	948
**Residence**			
Urban	64.9	82.7	1
Rural	60	77.7	3
**Region**			
Ashanti	60.7	85.5	922
Brong Ahafo	59.5	80.8	564
Central	64.6	84.9	479
Eastern	57.7	78.4	567
Greate Accra	57.6	74	470
Northern	67	69.8	699
Volta	54.6	78.5	451
Upper East	73.7	77.7	225
Upper West	71.4	79.1	152
Western	62.4	83.5	400
**Education**			
None	60.6	72	1,678
Primary	58.5	80.1	1,102
Middle/JSS	63.2	84.8	1,797
Secondary+	69.2	84.8	350
**Wealth quintile**			
Lowest	61.6	74.1	1,074
Second	57.3	75.5	1,061
Middle	61.2	81.6	975
Fourth	62.1	82.6	983
Highest	67.6	85	835
**Total**	**61.7**	**79.4**	**4,928**

**Table 4:** **Tetanus toxoid immunization during pregnancy for the last live birth or stillbirth in the five years preceding the survey by religion and ethnicity**

**Background characteristic**	**Had tetanus toxoid injection**	**No tetanus toxoid injection**	**Don’t know**	**Total**	**Number of births**
**Religion**					
Catholic	88.7	10.8	0.3	100	694
Protestant	90	10	0	100	80
Methodist	93.4	6	0.6	100	348
Presbyterian	91.2	8.8	0	100	317
Pentecostal/charismatic	90.2	9	0.8	100	1404
Other Christian	87.8	11.8	0.4	100	836
Moslem	92.5	7.5	0	100	896
Traditional/spiritualist	72.5	27.5	0	100	207
No religion	85.7	14.3	0	100	293
Other	100	0	0	100	1
**Ethnicity**					
Akan	91.8	7.7	0.6	100	2248
Ga/Dangme	85.9	13.9	0.2	100	404
Ewe	85.6	14.3	0.2	100	644
Guan	86.6	13.4	0	100	119
Mole-Dagbani	88.9	11.1	0	100	548
Grussi	91.9	7.7	0.4	100	246
Gruma	84.3	15.5	0.3	100	343
Hausa	95.2	4.8	0	100	62
Other	88.6	11.4	0	100	463
**Total**	**89.3**	**10.3**	**0.3**	**100**	**5077**

Note: Total includes 149 women with missing information on tetanus toxoid immunization.

### Inequities in access and use of delivery care services

**Figure 5:**
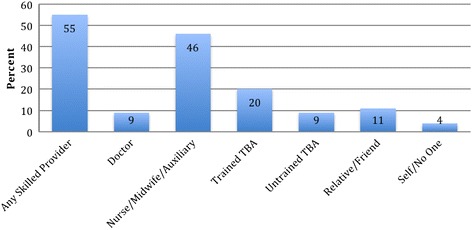
**Per cent distribution of most recent live or stillbirth in the five years preceding the survey by person providing assistance during delivery.**

Figure 5 shows that skilled providers (i.e. a doctor, nurse/midwife or auxiliary midwife) delivered just a little over one-in-two births (55%) in Ghana. However, this national statistic tells little about the fact that pervasive access inequities exist between women of different socio-demographics as can be observed in Tables 5 and 6. For instance, the number of births to women in the Greater Accra, Ashanti and Western regions that were delivered in health facilities with a medical doctor in attendance was twice the number of births to women in the Northern and Upper regions (Table 5). Similarly, 88% of births to women with at least secondary education occurred in a health facility, compared with 31% of births to women with no education, while 92% of women in the highest wealth quintile had institutional deliveries, compared with 27% of women in the lowest wealth quintile (Table 6). Also more births to women living in urban areas took place in a health facility compared to births to women living in rural Ghana. For instance, four-in-five births in Greater Accra were delivered in a health facility, compared with one-in-four births in the Northern Region.

**Table 5:** **Per cent distribution of live or stillbirth in the five years preceding the survey by person providing assistance during delivery**

**Background characteristic**	**Doctor**	**Nurse/midwife/auxiliary midwife**	**Trained traditional birth attendant**	**Untrained traditional birth attendant**	**Relative/Friend**	**No one**	**Other**	**Missing**	**Total**	**Percent delivered by a skilled provider**	**Percent delivered by CS**	**Number of births**
**Birth outcome**												
Live birth	8.9	46.1	20.3	9.5	11.2	3.7	0	0.3	100	55	6.3	4847
Stillbirth	28.6	40.8	11.5	1.6	7.5	5.3	2.8	2	100	69.3	18.9	81
**Age of mother at birth**												
<20	8.6	46.5	22.2	9.1	11.7	1.9	0	0	100	55.1	6.5	534
20–34	9.3	47.4	19.6	9.4	11	3.1	0	0.2	100	56.7	6.6	3391
35–49	9.2	41.1	21	9.4	11.5	6.8	0.2	0.9	100	50.2	6.3	1003
**Birth order**												
1	12.4	54.6	16.8	7.6	7	1.1	0	0.5	100	67	9.4	1051
2–3	9.2	49.2	19.5	8.6	11.3	2.1	0	0.1	100	58.4	6.4	1769
4–5	8.1	43.8	21.1	9.8	12.6	4.2	0.1	0.2	100	51.9	5.9	1161
6+	6.9	33.3	23.8	12.1	13.8	9	0.2	0.8	100	40.3	4.2	948
**Residence**												
Rural	5.4	33.8	27.4	13	15.1	4.8	0.1	0.3	100	39.2	4	3781
Urban	16.5	69.5	6.1	2.3	3.6	1.5	0	0.5	100	86	11.3	1683
**Education**												
None	4	28.3	25.8	16.6	18.5	6.1	0.1	0.5	100	32.4	3.4	1678
Primary	8.6	43.6	21.8	9.3	11.8	4.2	0.1	0.6	100	52.2	6.4	1102
Middle/JSS	11.8	60	16.3	4.1	5.9	1.8	0	0.1	100	71.7	7.9	1797
Secondary+	22.6	66.6	7.4	1.5	1.4	0.5	0	0	100	89.2	14.6	350
**Region**												
Ashanti	13.8	54.8	13.2	6.2	9	2.6	0.1	0.2	100	68.7	8.6	922
Brong Ahafo	6.3	50.6	18.2	7.9	11.1	5.6	0	0.3	100	56.9	5.1	564
Central	6.1	57.8	25	4.5	2.8	3.7	0	0.2	100	63.8	6.3	479
Eastern	8.8	48.9	22.2	8.3	8.2	3.6	0	0.1	100	57.7	8.4	567
Greater Accra	24.2	55.1	10.2	2.9	3.7	3.6	0	0.4	100	79.3	13.1	470
Northern	4.8	22.5	21	24.5	21	5.6	0.3	0.2	100	27.3	3.1	699
Volta	2.7	38.7	22.4	5.5	26.9	3.8	0	0.1	100	41.3	3.5	451
Upper East	2.2	45	31.6	9.7	9.3	1.5	0	0.8	100	47.1	2.4	225
Upper West	3.2	39	33.3	5.7	13.8	4.6	0	0.4	100	42.2	3.7	152
Western	10.5	43.1	26.3	12.8	4.4	1.5	0	1.5	100	53.7	6.1	400
**Wealth quintile**												
Lowest	3.2	25.2	26	17.6	21.5	6.3	0	0.2	100	28.4	2.7	1074
Second	4.6	32.3	28.8	13.3	15.3	5.1	0.3	0.2	100	36.9	3.1	1061
Middle	6.4	46.5	24.8	8.5	9.7	3.9	0	0.2	100	52.9	4.7	975
Fourth	12.5	63	13.7	3.5	5.3	1.5	0	0.6	100	75.5	8.7	983
Highest	22.1	69.6	3.8	1.6	1.4	1	0	0.5	100	91.8	15.4	835
**Total**	**9.2**	**46**	**20.1**	**9.4**	**11.2**	**3.7**	**0.1**	**0.3**	**100**	**55.2**	**6.5**	**4928**

**Table 6:** **Per cent distribution of most recent live or stillbirths in the five years preceding the survey by place of delivery**

**Background characteristic**	**Public health facility**	**Private health facility**	**Home**	**Other**	**Missing**	**Total**	**Percent delivered in a health facility**	**Number of births**
**Birth outcome**								
Live birth	42.9	11.1	45.4	0.4	0.1	100	54.1	4847
Stillbirth	61.5	6.3	26.1	4	2	100	67.9	81
**Age of mother at birth**								
<20	43.9	10.3	45.7	0.1	0	100	54.2	534
20–34	44.5	11.2	43.8	0.4	0.1	100	55.7	3391
35–49	38.8	10.8	49.3	0.7	0.4	100	49.6	1003
**Birth order**								
1	53.3	13.7	32.4	0.3	0.2	100	67.1	1051
2–3	46.3	10.6	42.8	0.1	0.1	100	56.9	1769
4–5	40.2	11.2	47.7	0.8	0.2	100	51.4	1161
6+	30.1	8.6	60.3	0.8	0.2	100	38.7	948
**Residence**								
Rural	31.6	6.6	61.2	0.5	0	100	38.2	3245
Urban	65.8	19.5	14.1	0.2	0.4	100	85.3	1683
**Education**								
None	25.7	5.5	68	0.6	0.2	100	31.2	1678
Primary	41.6	10.2	47.4	0.3	0.4	100	51.8	1102
Middle/JSS	56.6	14.2	28.7	0.4	0	100	70.8	1797
Secondary+	64	23.8	12.1	0	0	100	87.9	350
**Region**								
Ashanti	54.5	13.2	31.3	0.8	0.2	100	67.7	922
Brong Ahafo	43.1	13.6	42.8	0.4	0.1	100	56.7	564
Central	42.6	15.9	41.2	0.3	0	100	58.5	479
Eastern	47.9	10.4	41.4	0.1	0.1	100	58.3	567
Greater Accra	55	24	20.1	0.5	0.4	100	79	470
Northern	21.9	4.4	72.9	0.7	0.2	100	26.3	699
Volta	35.3	5.8	58.7	0	0.1	100	41.2	451
Upper East	45.8	0.9	53.4	0	0	100	46.6	225
Upper West	40.2	1	58.8	0	0	100	41.2	152
Western	43.8	9.4	45.8	0.5	0.5	100	53.2	400
**Wealth quintile**								
Lowest	24.3	2.7	72.7	0.2	0	100	27	1074
Second	30.2	5.6	62.8	1.2	0.1	100	35.8	1061
Middle	41.4	10.5	47.9	0.2	0	100	51.9	975
Fourth	58.3	16.3	24.6	0.4	0.4	100	74.6	983
Highest	68.6	23	8	0	0.4	100	91.6	835
**Total**	**43.3**	**11**	**45.1**	**0.4**	**0.2**	**100**	**54.3**	**4928**

Two other variables against which we assessed equity of access to skilled delivery care are religion and ethnicity. As shown in Table 7, the range for consultation of a health worker for prenatal or delivery care among religious groups is 50.9 percentage points; from a low of 24% among births to women of traditional African religious orientation to 75% among births to Pentecostal and Charismatic Christians. Overall, women professing traditional/spiritualist and Moslem religious faiths accessed and used less of health facility delivery services compared with Christians. As regards ethnicity, few Hausa (13%), Ga/Dangme (22%) and Akan (23%) women gave birth at home compared to women of the Ewe (44%), Guan (57%), Grussi (60%), Mole-Dagbani (61%) and Gruma (66%) ethnic extractions.

**Table 7:** Per cent distribution of most recent live or stillbirths in the five years preceding the survey by place of delivery according to religion and ethnicity*

**Background characteristic**	**Own’s home**	**Other home**	**Government hospital/polyclinic**	**Government health center**	**Government health post/clinic**	**Other public health facility**	**Private hospital clinic**	**Private maternity home**	**Other private health facility**	**Other**	**Total**	**Number of women**
**Religion**												
Catholic	39.3	5.3	30	9.4	4.3	0.1	6.8	3.9	0.4	0.4	100	694
Protestant	30	1.2	41.2	13.8	3.8	0	6.2	2.5	0	1.2	100	80
Methodist	28.7	5.5	35.9	10.1	4.6	0	9.5	5.2	0	0.6	100	348
Presbyterian	28.4	6.6	38.2	9.5	4.4	0.3	7.3	5.3	0	0	100	317
Pentecostal/Charismatic	25.4	8	36.3	9.5	4	0.1	9.6	5.9	0.9	0.3	100	1404
Other Christian	31.3	7.1	31.1	11.1	5.5	0	8.1	5.1	0.1	0.5	100	836
Moslem	47.5	2.5	27.6	8.4	2.5	0	7.3	3.9	0.1	0.3	100	895
Traditional/Spiritualist	76.3	5.3	6.3	6.8	2.9	0	1	0.5	0	1	100	207
No Religion	65.2	5.5	10.6	9.6	4.4	0	2.4	1.7	0	0.7	100	293
Other	0	0	100	0	0	0	0	0	0	0	100	1
**Ethnicity**												
Akan	23.3	7.2	36.8	10.8	5.4	0	9.7	5.9	0.5	0.4	100	2.248
Ga/Dangme	21.5	11.4	37.1	8.7	3.7	0.2	9.2	6.4	1.2	0.5	100	404
Ewe	43.5	5.4	28.3	8.2	2.8	0	6.4	5	0.2	0.3	100	644
Guan	57.1	9.2	19.3	2.5	2.5	0	5.9	1.7	0	1.7	100	119
Mole-Dagbani	60.7	2.4	20.3	7.5	2.6	0	3.8	2.2	0	0.5	100	547
Grussi	59.8	0.4	22.4	8.5	2.8	0	5.7	0.4	0	0	100	246
Gruma	66.8	2.9	15.5	9.3	1.5	0.3	2.6	0.9	0	0.3	100	343
Hausa	12.9	3.2	59.7	3.2	0	0	12.9	8.1	0	0	100	62
Other	44.3	4.5	23.5	11.9	5	0	6.5	3.7	0.2	0.4	100	463
**Total**	**37**	**5.9**	**30.5**	**9.6**	**4.1**	**0.1**	**7.6**	**4.6**	**0.4**	**0.4**	**100**	**5076**

*Total includes 148 women with missing information on place of delivery.

### Inequities in access and use of caesarean sections during delivery

Tables 5 and 8 contain information about access and use of caesarean section (CS) services among women who had a live or stillbirth in the five years preceding the survey. There are large disparities for this indicator too. Whereas the percentage of women delivering by CS is as high as 13.1% in the Greater Accra region for example, the Northern and Upper East regions recorded 3.1% and 2.4% respectively. Also whereas 11.3% of urban women used CS during their last birth in the five years preceding the survey, only 4% of rural women did (see Table 5). The differences in terms of wealth quintiles are also striking: 15.4% for the highest and 2.7% for the lowest. First time mothers are also more than twice (9.4%) likely to use deliver by CS compared to 6th order and above births (4.2%). There are also striking access differentials between different religious and ethnic groups. Whereas 15% of Presbyterian Christians accessed and used CS services, only 6% of women professing traditional/spiritualist religion did. Moslem women also accessed and used CS services less compared with their Christian counterparts. In terms of accessibility and utilization according to ethnic affiliation, 16% (the highest) of Ewe and only 6% (the lowest) of Gruma women delivered their last baby by CS.

**Table 8:** Per cent distribution of most recent live or stillbirth in the five years preceding the survey by whether delivery was caesarean section according to religion and ethnicity*

**Background characteristic**	**Delivery by CS**	**Other**	**Total**	**Number of births**
**Religion**				
Catholic	12.9	87.1	100	380
Protestant	11.1	88.9	100	54
Methodist	11.6	88.4	100	225
Presbyterian	14.5	85.5	100	207
Pentecostal/Charismatic	12.5	87.5	100	928
Other Christian	12.4	87.6	100	510
Moslem	10.9	89.1	100	440
Traditional/Spiritualist	5.7	94.3	100	35
No Religion	9.6	90.4	100	83
Other	0	100	100	1
**Ethnicity**				
Akan	12.4	87.6	100	1551
Ga/Dangme	12.6	87.4	100	269
Ewe	16	84	100	326
Guan	10.5	89.5	100	38
Mole-Dagbani	13.3	86.7	100	195
Grussi	7.2	92.8	100	97
Gruma	5.8	94.2	100	103
Hausa	11.5	88.5	100	52
Other	9.1	90.9	100	231
**Total**	**12.2**	**87.8**	**100**	**2862**

*Total excludes 2066 women with missing information on delivery by CS.

### Inequities in access to and use of postpartum care services

One intervention for which equity assessment can also be made in the context of Ghana’s user-fee exemption policy is postpartum care. Table 9 shows the percentage distribution by timing of first postnatal check-up among women with a live or stillbirth in the five years preceding the survey by birth outcome, place of delivery, residence, region, and wealth, while Table 10 shows the per cent distribution of whether postnatal care was received among women with a live or stillbirth in the five years preceding the survey, according to religion and ethnicity. About 76% (3 in 4) of women reported receiving post-delivery care for themselves and their babies during their last birth in the five years preceding the survey. However, a cursory analysis of Tables 9 and 10 reveals substantial differences and/ or inequities. Broadly, the differences in postpartum care access by demographic, socio-economic and residential background attributes mirror differences already seen for ANC and DC.

**Table 9:** **Per cent distribution of postnatal care provider among women with a live or stillbirth in the five years preceding the survey**

**Background characteristic**	**Received PNC**	**Received PNC from medically trained provider**	**Doctor**	**Nurse/midwife/auxiliary midwife**	**Trained traditional birth attendant**	**Untrained traditional birth attendant**	**Relative/Friend**	**Other**	**No one**	**Missing provider information**	**Missing PNC information**	**Total**	**Number of births**
**Birth outcome**													
Live Birth	75.9	54.8	12.9	41.8	11	4.7	5.2	0.2	23.9	0	0.2	100	4847
Stillbirth	73.3	61.9	35.8	26.1	5.9	0	5.5	0	24.8	0	2	100	81
**Place of delivery**													
Health facility	87	86.5	23.6	63	0.5	0	0	0	12.8	0	0.2	100	2675
Elsewhere	62.7	17.3	1.1	16.3	23.5	10.1	11.3	0.3	37.2	0.1	0.1	100	2244
**Had problems before, during or after delivery**													
No	74.9	52.5	11.1	41.4	11.6	5.2	5.3	0.1	25	0.1	0.1	100	3908
Yes	80	64.3	21.8	42.5	8.5	2.2	4.8	0.2	19.9	0	0.1	100	1011
**Residence**													
Rural	72.2	43.4	7.9	35.6	14.9	6.5	7.1	0.2	27.7	0.1	0.1	100	3245
Urban	82.7	76.9	23.7	53.2	3.4	0.9	1.4	0	16.6	0	0.6	100	1683
**Region**													
Ashanti	79.9	71	17.3	53.8	5.9	1.3	1.2	0.4	20	0	0.2	100	922
Brong Ahafo	90.9	63.4	8.2	55.1	14.8	4.5	8.2	0	8.7	0	0.3	100	564
Central	92.8	64.2	9.5	54.7	22.7	3.9	2	0	7	0	0.2	100	479
Eastern	66.1	57.4	14.6	42.8	5.7	1.5	1.3	0.2	33.4	0	0.4	100	567
Greater Accra	64.2	56.3	25.7	30.6	4.5	1.5	1.9	0	35.2	0	0.5	100	470
Northern	79.6	26.1	6.6	19.5	15.5	18.2	19.2	0.3	20.2	0.3	0.2	100	699
Volta	56	38.5	12	26.5	9.2	1.1	7.1	0	43.7	0	0.3	100	451
Upper East	77.1	66.8	4.7	62.1	10.3	0	0	0	22.9	0	0	100	225
Upper West	61.2	56.4	2	54.4	4.8	0	0	0	38.8	0	0	100	152
Western	72.7	50.6	21.6	29	14.9	5.8	1.4	0	26.8	0.1	0.5	100	400
**Wealth quintile**													
Lowest	65.8	35	5.4	29.7	12.3	8.4	9.7	0.2	34.2	0.1	0	100	1074
Second	73.8	41	6.4	34.6	17	6.8	8.6	0.3	26	0.1	0.2	100	1061
Middle	77.3	54.1	8.6	45.4	15.6	4.1	3.6	0	22.5	0	0.2	100	975
Fourth	80.2	69.8	20.2	49.6	6.3	1.6	2.3	0.2	19.2	0	0.6	100	983
Highest	84.3	81.5	29.6	51.9	1.7	0.9	0.3	0.1	15.1	0	0.5	100	835
**Total**	**75.8**	**54.9**	**13.3**	**41.6**	**11**	**4.6**	**5.2**	**0.1**	**23.9**	**0**	**0.3**	**100**	**4928**

**Table 10:** Per cent distribution of whether postnatal care was received among women with a live or stillbirth in the five years preceding the survey, according to religion and ethnicity*

**Background characteristic**	**Obtained PNC**	**No PNC**	**Total**	**Number of births**
**Religion**				
Catholic	77.7	22.3	100	694
Protestant	78.8	21.2	100	80
Methodist	85.3	14.7	100	346
Presbyterian	72.9	27.1	100	317
Pentecostal/charismatic	77.5	22.5	100	1402
Other Christian	75.2	24.8	100	836
Moslem	77.5	22.5	100	893
Traditional/spiritualist	65.7	34.3	100	207
No religion	63.1	36.9	100	293
Other	1	0	100	1
**Ethnicity**				
Akan	81.8	18.2	100	2244
Ga/Dangme	66.6	33.4	100	404
Ewe	66.5	33.5	100	644
Guan	81.4	18.6	100	118
Mole-Dagbani	78.3	21.7	100	548
Grussi	67.1	32.9	100	246
Gruma	66.8	33.2	100	343
Hausa	87.1	12.9	100	62
Other	76.6	23.4	100	461
**Total**	**76.1**	**23.9**	**100**	**5070**

*Total includes 142 women with missing information on PNC.

## Discussion

### Main results

This paper has attempted to assess the extent and nature of inequities in access to and use of maternal health services in Ghana after user-fee exemption for maternal health services. Results from our descriptive statistical analysis of survey data indicated that the implementation of the exemption policy in Ghana appeared to have been accompanied by marginal increases in the proportion of women who accessed and used antenatal, delivery, and postnatal care services from skilled health professional in a health facility setting. Our study has however revealed important discrepancies in access to and use of maternal health services that should not be underestimated. Except for ANC, our findings showed that the proportion of women who had access to delivery and post-delivery care was still low and even considerably lower for women of certain socio-demographic groupings such as the poor. For example 45% of births in the last five years before the survey took place at home without skilled attendance. Similarly, 45% of the women who gave birth during the same period did not receive any form of postpartum care. Thus our analysis has shown that substantial differences in access and service use characterized Ghana’s maternal health delivery system.

It is difficult to disentangle the effect of other factors than the user-fee exemption policy on the observed increases in service uptake. This is not only because the universal nature of Ghana’s user-fee exemption policy made it hard to conduct robust comparative analysis in our study, but also the data needed to conduct such analysis did not exist at the time of this research. Consequently, correlation here must not mean causation. Nevertheless, our findings support previous research in Ghana [11,29] and elsewhere [42,43] that found similar incremental changes in access to, and use of maternal health services following the abolition of user-fees for maternal health services.

Again, it is difficult to make any judgement about the relationship between Ghana’s free maternal health policy and the observed inequities in access to and use of maternal health services from our study. It is plausible that access inequities improved from an even more inequitable distributive baseline following the implementation of the policy or vice versa. The lack of relevant comparable data before the introduction of the policy did not permit this hypothesis to be further explored in our research. This notwithstanding, our findings are consistent with previous studies in Mali [20] and Kenya [22] which found that while user-fee exemptions removed important financial barriers, they were insufficient to ensure equal access to maternal health service.

That inequities in skilled care services accessibility and utilization exist across different sub-population groups in Ghana is worrying. It is worrying because Ghana’s user-fee exemption policy was intended to be universal. In practice, as our findings demonstrated, many women continue to deliver their babies at home or outside the provided government and non-government healthcare facilities without skilled care. The discrepancies in access to and use of maternal health services among women from different socio-economic backgrounds that we observed in our study should not be overlooked.

Our findings indicate important spatial inequities in access to and use of all components of maternal health services. For instance, 38% more women in the best performing geographic region (Central Region) than the worst (Upper East Region), accessed and used all skilled ANC, DC, PNC services in the five years preceding the survey. This utilisation differential could be related to a number of factors, including differences in coverage of maternal health services. In fact, those women most likely to give birth at home without skilled attendance or with a TBA came from geographic regions such as Upper West and East, Northern and Volta that generally suffer the worse forms of multiple deprivation including wealth, knowledge and health [44]. This deprivation in and of itself could put women from these impoverished regions in a particularly disadvantaged position in terms of their ability to get formal education, earn a decent income and access healthcare. In terms of policy, our findings here would indicate the need to direct more efforts and interventions towards those regions where access levels are low.

Wealth-related inequities were also documented. More women (53%) in the highest wealth quintile than women in the lowest accessed and used all components of skilled ANC, DC, PNC services in the five years preceding the survey. In general, skilled attendance at birth, delivery in a health facility, use of caesarean section (CS) during childbirth, and post-delivery services all indicated gradients that were in favour of the wealthiest. Less surprisingly, women in the poorest wealth quintile used more unskilled home delivery services offered by TBAs. In the context of Ghana where maternal health services are provided free at the point of delivery, poverty, unavailability of maternal health services, high transportation costs, difficulties with arranging appropriate transportation to seek care, as well as other opportunity and social costs associated with maternal health seeking, might explain the rich-poor gap in service accessibility and utilisation. Addressing the rich-poor gap in access to maternal health services could therefore be essential for achieving the maternal health MDG targets.

Education-related inequities in the rate of access and use of maternal health services were also observed. For example, 48% more women with at least secondary education than those with no formal education accessed and used all skilled ANC, DC, PNC services. Indeed, maternal education has been found to be positively associated with access and use of many of the elements of skilled maternity care such as delivering in a hospital [45,46]. Influences of maternal education on maternal healthcare access can be effected in several ways, including improving the ability of women of reproductive age to produce good maternal health outcomes without even relying on health services by influencing their reproductive behaviours such as contraceptive use, increasing women’s use of maternity care services through improved knowledge, attitude and practice, empowering women to be able to leverage decision-making power regarding reproductive choices and access to birth services within the household and community [47]. Our findings here would therefore indicate the need for improvement in women’s education up to at least secondary level in order to bridge the equity gap and improve access to essential maternal health services.

We also observed important urban-rural inequality in access to and use of maternal health interventions. For instance, 40% more urban women than rural women accessed and used all skilled ANC, DC, PNC services in the five years preceding the survey. This is consistent with previous studies in Ghana [48]. It might be difficult in the current study to identify the exact mechanisms by which rural-urban inequities are effected. However, we believe these access inequities could partly be linked to a number of supply-side factors, whereby there is urban-bias in the availability of, quality of, and ease of access to, maternal health services. This is more likely to be so because Ghana is known to have marked rural-urban disparity in health infrastructure [49]. It could also partly be because there is a high concentration of the better-educated and economically empowered women in urban areas than in rural areas. As discussed above, both education and wealth could contribute to enabling more urban women than rural women, to access maternal health services. In this regard, we think the practice of concentrating health facilities and resources in urban areas in Ghana need to change to ensure equity in access and to mitigate the distance barrier for rural women. In rural areas, we recommend the establishment of more maternal health clinics within reasonable distance to facilitate equitable access.

Our study has also revealed important access inequities between different religious groups. For example, whereas 15% of Presbyterian Christians accessed and used CS services, only 6% of women professing traditional/spiritualist religion did for their last birth in the five years preceding the survey. Moslem women also accessed and used CS services less compared with their Christian counterparts. That differences in religious affiliation influenced accessibility to, and utilisation of maternity care services in Ghana bear resonance with other previous studies. In Ghana, Addai [46] found that the range for consultation of a health worker for prenatal care among religious groups was 12.8 points, from 10.3% among women of traditional African religious orientation to 23.1% among Catholic and Protestant women. Gyimah *et al* [48] also found that Moslem and Traditional women were less likely to use maternal health services in Ghana compared with Christians. Elsewhere in India one study also found that Muslim women were less likely to use reproductive, sexual, and maternal health services compared with Christians [50]. It is difficult to tell from our study how issues in Muslim culture or Traditional African religious beliefs act as barriers to use of maternal services; neither can we exactly explain why Catholic women for example, patronise more maternal health services than Muslim women or women with Traditional African religious orientation. For this reason, we support Gyimah *et al* [48] call for more qualitative research into aspects of religious affiliation that discourage access and use of maternal health services.

Lastly, fewer women from majority ethnic groups such as the Akan (23%) and Ga/Dangme (22%) were found to have given birth at home compared with women from minority ethnic groups such as the Ewe (44%), Guan (57%), Grussi (60%), Mole-Dagbani (61%) and Gruma (66%). Being in a minority ethnic group has been found to be a barrier to access to and use of maternal health services [51]. One previous study in Ghana found that while almost equal proportions of women of the Ga-Adangbe, Fante and Akan ethnic groups used the hospital or health facility as place of delivery, lower proportions are observed for women of Ewe, Guan, Gruma and other minority ethnic backgrounds [46]. Indigenous women in Guatemala [52] and Mexico [53], and ethnic minorities in China [54] have been found to be less likely to have skilled attendance at delivery.

It is not possible to say from our study why minority ethnic women had less access to care compared with majority ethnic women, neither are we able to determine whether belonging to a majority group such as the Akan or a minority group such as the Gruma automatically implies more access and less access respectively. We believe that more qualitative research is needed to explore these issues. We however think these access differentials could be explained by the fact that women from minority ethnic groups are more likely to suffer discrimination and abuse upon entry into the healthcare system. For this reason, the social imperatives for such women to avoid the formal healthcare system simply are powerful. Elsewhere in Bangladesh, Schuler and colleagues [25] have documented a similar phenomenon. Naturally, differences in discrimination introduces differential costs of accessing care for different people, and this violates the second requirement of equity of access which argues for a situation in which individuals or groups face equal or equivalent access and costs of utilization for equal or equivalent needs. In this regard, it might be useful for policymakers in Ghana to take urgent steps to develop comprehensive need-based targeting and resource allocation formula that can target more resources and services towards minority populations.

### Methodological considerations

Our findings and recommendations in this paper should be read against the backdrop of certain potential limitations. Our study design could potentially have made it impossible to isolate the effects (positive or negative) of the user-fee exemption policy on access and inequity in access. Ideally, a pre-post evaluation design would have been most appropriate for investigating the impact of implementing the exemption policy on skilled care services accessibility and utilization in Ghana. In particular, pursuing a counter-factual analysis would have been the best approach in determining whether the policy actually increased access and improved equity in access and use of services. That is, what would have happened to access and equity in the absence of the policy during the period under consideration (2003–2007)? However, because the policy was implemented nation-wide, such an analysis was not feasible as there are no appropriate comparison groups against which a comparative assessment can be made. Unfortunately, both the design and remits of this research failed to extend to an evaluation and statistical estimation or quantification of how much of either the increases in overall utilisation levels or the differences in utilisation among different groups is directly attributable to the fee exemption policy.

Also, secondary data come with its own strengths and weaknesses, and the GMHS data we used is no exception. At the commencement of our research, this data set was one of the best readily available, and up-to-date databases on maternal healthcare access in Ghana. The main strengths of the data are the large sample size and its representativeness of the population. Both of the two attributes are known to increase precision of estimates of study sub-groups [55]. However, given that the data is almost six years old, its relevance for capturing current access and utilisation levels and patterns may be diminished.

## Conclusion

Like many health policies that aim to address financial barriers to healthcare access, Ghana’s user-fee exemption policy was based on an assumption that all women would avail themselves to access and use maternal health services if only these services became more affordable [18]. The findings in this paper suggest that although removing user-fees has the potential to improve access to skilled care, it is neither sufficient nor appropriate for eliminating inequities in access in some contexts. Our findings and discussion clearly indicate that differences in women’s *socio-economic status*, as represented by differentials in educational attainment, wealth, type of residence, geographical region, religious affiliation, and ethnic background play a crucial role in the continued practice of maternal health services under-utilisation, and unequal access to skilled care and homebirth. That differences in women’s socio-demographic attributes influence their ability to access and use maternal health services in Ghana clearly violates one of the fundamental requirements for equity of access, namely that non-medical or non-biological features of individuals or groups should not determine their access to healthcare. At the same time, most of these socio-economic status variables lie outside the confines of the healthcare system. This suggests that if equity in access to maternal health is to be achieved in Ghana, the policy debates on user-fees removal ought to proceed beyond increases in service utilisation towards exploring who continues to remain excluded from access to maternal care services following user-fee exemption, and how best to address the multiplicity of access and utilisation barriers other than money that might prevent some women from seeking care. In this regard, we believe a concerted multi-sectorial approach is needed to tackle the social determinants of health as well as address the wider issue of economic, social and political disadvantage, including raising the educational attainment and living conditions of disadvantaged women, improving the availability, distribution and quality of physical health infrastructure, and increasing the quantity and capacity of human resources for maternal health.
